# Systematic Review of Pediatric Brain Tumors in Neurofibromatosis Type 1: Status of Gene Therapy

**DOI:** 10.7759/cureus.27963

**Published:** 2022-08-13

**Authors:** Sonu Thomas, Viktoriya Bikeyeva, Ahmed Abdullah, Aleksandra Radivojevic, Anas A Abu Jad, Anvesh Ravanavena, Chetna Ravindra, Emmanuelar O Igweonu-Nwakile, Safina Ali, Salomi Paul, Shreyas Yakkali, Sneha Teresa Selvin, Pousette Hamid

**Affiliations:** 1 Medicine, California Institute of Behavioral Neurosciences & Psychology, Fairfield, USA; 2 Internal Medicine, California Institute of Behavioral Neurosciences & Psychology, Fairfield, USA; 3 Behavioral Neurosciences and Psychology, California Institute of Behavioral Neurosciences & Psychology, Fairfield, USA; 4 General Surgery, California Institute of Behavioral Neurosciences & Psychology, Fairfield, USA; 5 Neurology, California Institute of Behavioral Neurosciences & Psychology, Fairfield, USA

**Keywords:** von recklinghausen’s disease, neurofibromatosis type 1, gene therapy, glioblastoma, brain stem glioma, optic pathway glioma, brain tumor

## Abstract

As oncology practice is rapidly shifting away from toxic chemotherapy, gene therapy provides a highly specific therapeutic approach for brain tumors. In this systematic review, we investigate gene therapy’s status in pediatric brain tumors and future recommendations. The search was conducted systematically using PubMed, Cochrane, Google Scholar, and ClinicalTrials.gov databases. The field search used in the process was selected based on the keywords and Medical Subject Headings (MeSH), depending on the database used. We included cases of neurofibromatosis type 1 (NF1) brain tumors in all age groups with the additional inclusion of English language, free full text, articles published within the last 20 years, randomized controlled trials (RCTs), observational studies, systematic reviews, and meta-analyses. We excluded case reports, case studies, and editorials. The search identified a total of 1,213 articles from the databases. We included 19 studies with 16 narrative reviews, one systematic review, and two randomized clinical trials with 43 patients. After reviewing all data in the articles, we found that gene therapy can improve standard treatment efficacy when used as adjuvant therapy. It can be used to overcome barriers such as chemotherapy resistance by downregulating resistance genes. It is associated with mild toxicity when compared with other available treatment options, but given the overall poor prognosis in pediatric brain tumors, further studies are warranted.

## Introduction and background

As oncology practice is rapidly shifting away from toxic chemotherapy, gene therapy provides a highly specific therapeutic approach for brain tumors. This treatment is rapidly evolving to deliver specific therapeutic genes or oncolytic viruses to eliminate the tumor, which can lead to tumor cell death and increased immune responses to tumor antigens, and disruption of the tumor microenvironment (TME), including angiogenesis/neovascularization inhibition [1]. Oncolytic virotherapy (OV), suicide gene therapy, tumor suppressor gene delivery, immunomodulatory strategies, and gene target therapies are the various types of gene therapies. Gene therapy delivery methods include direct delivery of therapeutic genes into the tumor site, which include virus-mediated adenovirus, herpes simplex virus-1 (HSV-1), adeno-associated virus-2, nonviral vector-based nanoparticles, liposomes, and micelles. Neural stem cells and mesenchymal stem cells are tumor-tropic cell carriers that express therapeutic gene(s) in the tumor site. PH-sensitive drug release, pH-sensitive liposomal carriers, and stimuli-responsive particles are examples of intelligent carriers [2].

According to the National Brain Tumor Society (NBTS), approximately 700,000 Americans have been diagnosed with a primary brain tumor, with 63% being benign and 37% being malignant. Brain tumors were the 10th leading cause of death in 2020 [3]. The pediatric brain tumors associated with neurofibromatosis type 1 (NF1) are optic pathway gliomas (OPGs), brain stem gliomas, glioblastomas, and pilocytic astrocytoma [4]. Brain and central nervous system (CNS) tumors have been reported in approximately 20% of patients with NF1 and are typically discovered in childhood. Optic pathway gliomas (OPGs) account for approximately 70% of all CNS tumors in children with NF1, while brain stem glioma accounts for approximately 17% of all CNS tumors [5]. Despite recent advances in surgery, radiotherapy, and chemotherapy, brain tumor treatment regimens have only a limited impact on long-term disease control [6]. The price of the cure is frequently unacceptable, and it includes acute and chronic organ toxicity, resistance to therapy, and more concerning, an increased risk of secondary malignancy. As a result, new strategies are required to improve overall survival and reduce treatment-related morbidity [7]. To tackle this situation, a better understanding of the functions and control of genes was needed, which paved the way for the development of gene therapy in the last decades [6].

The current study aims to provide an advance in gene therapies for pediatric brain tumors with neurofibromatosis type 1. This includes different genomic alterations seen in brain tumors and gene delivery systems comprising viral and nonviral delivery platforms along with suicide/prodrug, oncolytic, cytokine, and tumor suppressor-mediated gene therapy approaches. Finally, we discuss the results of gene therapy-mediated human clinical trials and highlight the progress, prospects, and remaining challenges of gene therapies aiming at broadening our understanding and highlighting the therapeutic approach for pediatric brain tumors.

## Review

Methods

This systematic review was performed in March 2022 using the Preferred Reporting Items for Systematic Reviews and Meta-Analyses (PRISMA) 2020 guidelines [8].

Eligibility Criteria

The inclusion criteria were cases of neurofibromatosis type 1 brain tumors in all age groups with the additional inclusion of English language, free full text, articles published within the last 20 years, randomized controlled trials (RCTs), observational studies, systematic reviews, and meta-analyses. We excluded case reports, case studies, and editorials.

Databases and Search Strategy

The search was conducted systematically using PubMed, Cochrane, Google Scholar, and ClinicalTrials.gov databases by the first and second authors separately. Table 1 summarizes the search strategy.

**Table 1:** Search strategy in detail MeSH: Medical Subject Headings

Databases	Keywords/MeSH	Filters	Search results
PubMed	Keywords: brain tumor, optic pathway glioma, brain stem glioma, glioblastoma, gene therapy, neurofibromatosis type 1, von Recklinghausen’s disease; MeSH: brain tumor OR optic pathway glioma OR brain stem glioma OR glioblastoma AND Gene therapy AND Neurofibromatosis type 1 OR von Recklinghausen’s disease AND Brain tumor (“Brain Neoplasms/therapy” (Majr)) OR “Brain Neoplasms/therapy” (Mesh:NoExp) AND Gene therapy ((“Genetic Therapy/methods” (Majr) OR “Genetic Therapy/statistics and numerical data” (Majr) OR “Genetic Therapy/therapy” (Majr))) OR (“Genetic Therapy/methods” (Mesh:NoExp) OR “Genetic Therapy/statistics and numerical data” (Mesh:NoExp) OR “Genetic Therapy/therapy” (Mesh:NoExp)) AND Neurofibromatosis type 1 (“Neurofibromatosis 1/classification” (Majr) OR “Neurofibromatosis 1/genetics” (Majr) OR “Neurofibromatosis 1/statistics and numerical data” (Majr) OR “Neurofibromatosis 1/therapy” (Majr)) OR (“Neurofibromatosis 1/classification” (Mesh:NoExp) OR “Neurofibromatosis 1/genetics” (Mesh:NoExp) OR “Neurofibromatosis 1/statistics and numerical data” (Mesh:NoExp) OR “Neurofibromatosis 1/therapy” (Mesh:NoExp)); advanced search: ((Brain tumor OR optic pathway glioma OR brain stem glioma OR glioblastoma) AND (gene therapy)) AND (Neurofibromatosis type 1 or von Recklinghausen’s disease)	Last 20 years, free full text, English language	578
Google Scholar, Cochrane	Keywords: brain tumor, optic pathway glioma, brain stem glioma, glioblastoma, gene therapy, neurofibromatosis type 1, von Recklinghausen’s disease	Last 20 years, English language	587 and 48, respectively

Results

**Figure 1:**
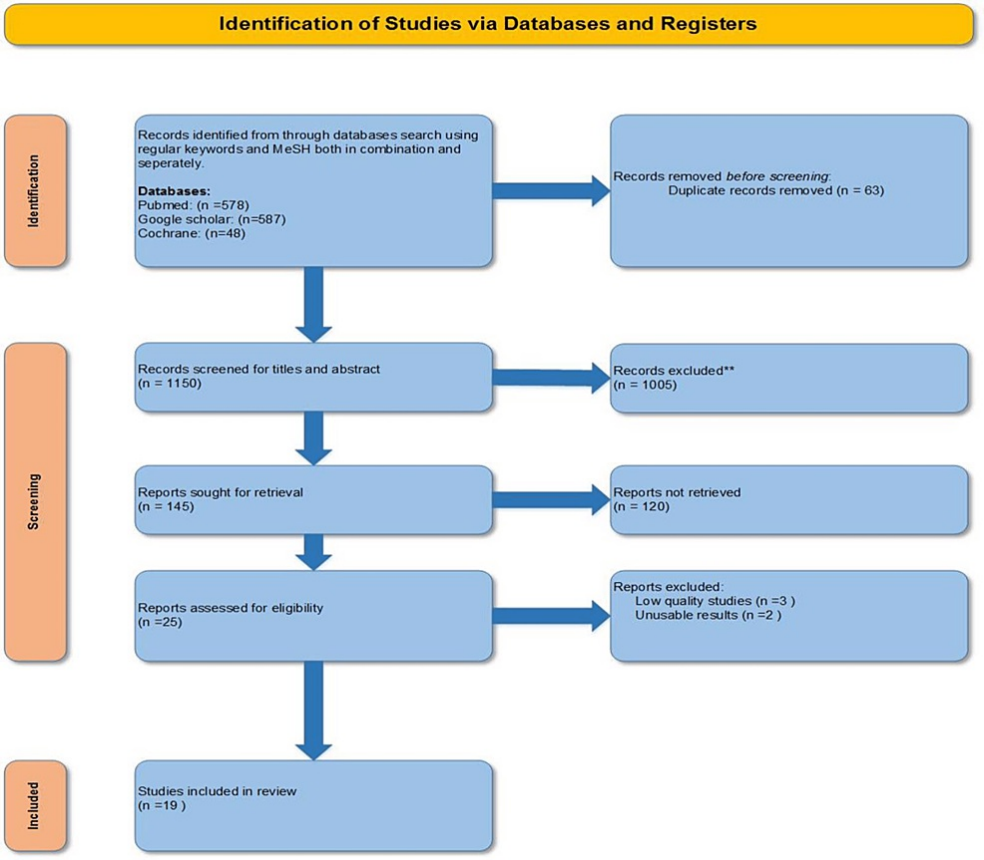
PRISMA flowchart showing study selection PRISMA: Preferred Reporting Items for Systematic Reviews and Meta-Analyses [8]; MeSH: medical subject heading

The search identified a total of 1,213 articles from the databases. EndNote is used to remove duplicated articles. The remaining articles were screened manually by the first and second authors. A total of 145 articles from databases were sought for retrieval, and 25 articles from the databases were retrieved and sent for quality appraisal. The articles were assessed for quality by the first two authors separately using tools depending on the study type: Cochrane Collaboration Risk of Bias Tool (CCRBT) for randomized control trials [9], Scale for the Assessment of Narrative Review Articles 2 (SANRA 2) for narrative reviews [10], and Assessment of Multiple Systematic Reviews (AMSTAR) for systematic reviews and meta-analyses [11]. Nineteen studies included in the review were scored above 70% (Figure 1).

Table 2 shows the result of the summary of the quality assessment of narrative reviews by authors.

**Table 2:** Result summary of the quality assessment of narrative reviews by authors

Author, year	Justification of the article’s importance for the readership	Statement of concrete aims or formulation of questions	Description of the literature search	Referencing	Scientific reasoning	Appropriate presentation of data	Sum score
Iwami et al., 2010 [6]	2	2	0	2	2	2	10
King et al., 2005 [12]	2	2	1	2	2	2	11
Kane et al., 2015 [13]	2	2	1	2	2	2	11
Natsume et al., 2008 [14]	2	1	0	2	2	2	9
Okura et al., 2014 [15]	2	2	0	2	1	2	9
Li et al., 2021 [16]	2	1	0	2	2	2	9
Curtin et al., 2005 [17]	2	2	0	2	2	2	10
Biagi et al., 2003 [7]	2	2	0	2	2	2	10
Lucifero et al., 2020 [18]	2	1	2	2	2	1	10
Candolfi et al., 2009 [19]	2	2	0	2	2	2	10
Castro et al., 2011 [20]	2	2	0	2	2	2	10
Kroeger et al., 2010 [21]	2	2	0	2	2	2	10
Murphy et al., 2013 [1]	2	2	0	2	2	2	10
Fulci et al., 2007 [22]	2	2	0	2	2	2	10
Assi et al., 2012 [23]	2	2	0	2	2	2	10
Marsh et al., 2013 [24]	2	2	0	2	2	2	10

In the study by Immonen et al., compared to controls (n = 7 patients), there is a substantial rise in the mean number of tolerated O6-benzylguanine (O6BG)/temozolomide (TMZ) cycles (P = 0.05) with gene therapy. The median progression-free survival was nine months, and the overall survival was 20 months. The study revealed delayed tumor growth at lower cumulative TMZ doses in the study patients compared to those who received standard regimens, concluding that this supports the chemoprotective effect of gene therapy when used in combination with O6BG and TMZ [25]. In the study of Adair et al., treatment of adenovirus-mediated herpes simplex virus thymidine kinase (AdvHSV-tk) resulted in a clinically and statistically significant increase in mean survival from 39.0 ± 19.7 (standard deviation) to 70.6 ± 52.9 weeks (P = 0.0095). From 37.7 to 62.4 weeks, the median survival time also increased, and treatment was well tolerated. The authors concluded that AdvHSV-tk gene therapy with ganciclovir (GCV) could be a promising new treatment [26]. Table 3 summarizes the risk of bias in randomized controlled trials using the Cochrane Collaboration Risk of Bias Tool (CCRBT).

**Table 3:** Risk of bias summary of randomized controlled trials using the Cochrane Collaboration Risk of Bias Tool (CCRBT)

Author, year	Random sequence generation	Allocation concealment	Selective outcome reporting	Other bias	Blinding of participants and personnel
Immonen et al., 2004 [25]	Low risk	Low risk	Low risk	Low risk	Unclear risk
Adair et al., 2014 [26]	Low risk	Unclear risk	Low risk	Low risk	Low risk

Table 4 summarizes the result of critical appraisal for systematic reviews and meta-analyses by review authors.

**Table 4:** Result summary of critical appraisal for systematic reviews and meta-analyses by review authors Yes: one point; no: zero point

Author, year	#1	#2	#3	#4	#5	#6	#7	#8	#9	#10	# 11	Total
Lucifero et al., 2021 [27]	Yes	No	Yes	Yes	No	Yes	Yes	Yes	Yes	No	Yes	8

Discussion

Brain tumors account for 21% of childhood malignancies and are the primary cause of solid tumor cancer death in children. Children affected with neurofibromatosis type 1 (NF1) are prone to optic pathway gliomas, brain stem gliomas, glioblastomas, and pilocytic astrocytoma. Two-thirds of gliomas are found in the optic pathway, with the brain stem, cerebellum, cerebral hemispheres, and subcortical structures accounting for the remaining locations. Chemotherapy is used to treat clinical progression, but surgery and radiation are difficult to use in the case of NF1-associated optic pathway gliomas since surgical resection is usually unachievable due to the tumor’s position. Radiation is not suggested for children with NF1 because of the possibility of secondary tumors (glioma and malignant peripheral nerve sheath tumors) in the context of this tumor predisposition syndrome, as well as the risk of late neurocognitive sequelae in children. Vincristine and carboplatin are used in first-line optic pathway glioma treatment. Vinblastine, vinorelbine, and temozolomide are the second-line chemotherapy options [28].

Overall, five-year survival rates for children less than 15 years of age are currently around 75% and 77% for those aged 15-19. Despite these advancements in treatment, a considerable number of individuals continue to be resistant to typical treatments. Acute and chronic organ damage, as well as an increased risk of secondary malignancy, are all disadvantages. Successful glioma treatment is hampered by ineffective medication distribution across the blood-brain barrier (BBB), an immunosuppressive tumor microenvironment (TME), and the development of drug resistance. Because gliomas are caused by the accumulation of genetic changes over time, gene therapy, which allows for the altering of the genetic makeup of target cells, appears to be a viable way to overcome the challenges that existing therapeutic strategies face [7].

**Figure 2:**
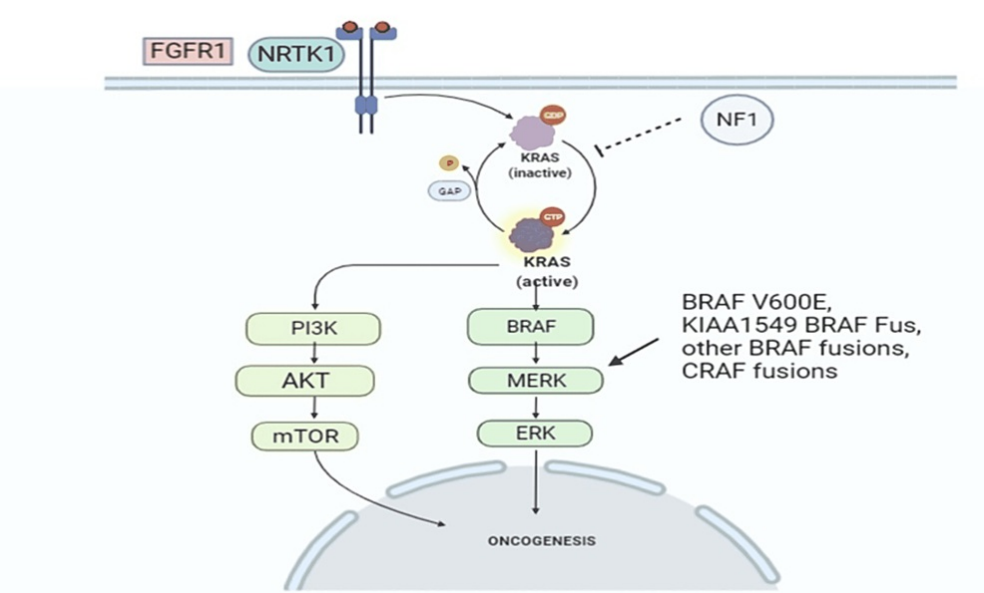
Pathways involved in oncogenesis FGFR1: fibroblast growth factor receptor 1; NRTK1: neurotrophic receptor tyrosine kinase 1; GTP: guanosine triphosphate; KRAS: Kirsten rat sarcoma virus; GDP: guanosine diphosphate; NF1: neurofibromin; PI3K: phosphoinositide 3 kinase; AKT: AK strain transforming; mTOR: mammalian target of rapamycin; ERK: extracellular signal-regulated kinases; MAPK: mitogen-activated protein kinase; RAF: rapidly accelerated fibrosarcoma gene; BRAF: proto-oncogene B-RAF; CRAF: proto-oncogene c-RAF; BRAF V600E: V600E is a mutation of the BRAF gene in which valine (V) is substituted by glutamic acid (E) at amino acid 600; KIAA1549-BRAF fusion: BRAF fusion involving the KIAA1549 gene This figure was originally created by the author.

Figure 2 explains the pathways involved in oncogenesis. By converting the active form of guanosine triphosphate (GTP)-bound Kirsten rat sarcoma virus (KRAS) to its inactive, guanosine diphosphate (GDP)-bound state, neurofibromin directly suppresses KRAS activation. Mitogen-activated protein kinases (MAPKs) and extracellular signal-regulated kinases 1 and 2 (ERK1 and ERK2) are activated by GTP-bound KRAS. The activation of rapidly accelerated fibrosarcoma gene (RAF)/MAPK causes transcription and cell proliferation to increase. Unchecked KRAS activation can also result in the cross-activation of the phosphoinositide 3 kinase (PI3K)-mammalian target of rapamycin (mTOR) pathway, which is critical for cell proliferation and survival. GTP-bound KRAS, for example, can bind and activate PI3K, resulting in survival and proliferation effects via AK strain transforming (AKT) and mTOR activity. As a result, neurofibromin deficiency can cause disease in a variety of ways. In gliomas, the KRAS, PI3K/phosphatase and tensin homolog (PTEN)/AKT pathways and neurogenic locus notch homolog protein (NOTCH) signaling are linked to cancer cell proliferation [29].

Glioblastoma Multiforme (GBM)

Complete resection of GBM is virtually impossible due to its intrusive nature and sensitive location. The current standard of care is a maximum surgical resection followed by radiation and temozolomide chemotherapy; however, the median survival time is still fewer than 15 months. This necessitates the creation of gene therapy, which delivers oncolytic viruses to the tumor in a precise manner to destroy it and lead to tumor cell death as well as increased immune responses to tumor antigens and disturbance of the tumor microenvironment, including angiogenesis/neovascularization inhibition [1]. The common gene targets that are mutated or upregulated in glioblastoma are neurofibromin, epidermal growth factor receptor (EGFR), phosphate and tensin (PTEN) homolog, platelet-derived growth factor (PDGF) receptor-alpha, isocitrate dehydrogenase-1 (IDH1), and tumor suppressor p53. GBM is a suitable candidate for gene therapy for several reasons: tumors remain within the brain with only rare metastases to other tissues; most cells in the brain are postmitotic, which allows for precise targeting of dividing tumor cells; tumors are often accessible neurosurgically for vector administration; sophisticated imaging paradigms are available; and standard therapies are minimally successful.

Delivery Methods for Gene Therapy

Table 5 summarizes the advantages, limitations, and clinical trials of the viral vectors used for gene therapy.

**Table 5:** Advantages, limitations, and clinical trials of the viral vectors used for gene therapy HSV-tk: herpes simplex virus thymidine kinase; TOCA511: retroviral replicating vector that selectively infects cancer cells and delivers cytosine deaminase; shRNA - short hairpin ribonucleic acid; MGMT: methylguanine methyltransferase; USA: United States of America; sh-SirT1 lentivirus: a lentivirus vector silencing sirtuin (silent mating type information regulation 2 homolog) 1; miR-100 lentivirus: a lentivirus vector with microRNA transfer; Ad: adenovirus; Adv-tk: adenovirus-mediated herpes simplex virus thymidine kinase; DNA: deoxyribonucleic acid; GAS1: growth arrest-specific 1; PTEN: phosphatase and tensin homolog; recombinant, SCH-58500: replication-deficient adenoviral vector containing the cloned human wild-type tumor suppressor gene p53; HSV1716: replication restricted oncolytic herpes simplex virus with antitumor effects in multiple cell lines; C134: genetically engineered herpes simplex virus; G207: neuroattenuated, replication-competent, recombinant herpes simplex virus-1; rQNestin34.5v.2: an oncolytic viral vector made from the herpes simplex virus type 1

Viral vector	Agent	Clinical trial number and phase	Advantages	Disadvantages	
Retrovirus	HSV-tk	NCT00001328, phase 1	Transfer to dividing cells, sustained expression of the vector	Elicit immune response, risk of insertion, low transfection rate in vivo, unable to transfect nondividing cells	
	TOCA511 (vocimagene amiretrorepvec) - retroviral replicating vector (RRV) that selectively infects cancer cells and delivers cytosine deaminase (CD)	NCT02414165, phase II/III			
Lentivirus	shRNA lentivirus		More stable and less prone to insertion mutation		
	MGMT gene	Case Western Reserve University, USA, phase I			
	sh-SirT1 lentivirus - a lentivirus vector silencing sirtuin (silent mating type information regulation 2 homolog) 1				
	miR-100 lentivirus - a lentivirus vector with microRNA transfer				
	GAS1-PTEN lentivirus				
Adenovirus	SCH-58500 - recombinant, replication-deficient adenoviral vector containing the cloned human wild-type (normal) tumor suppressor gene p53	NCT00004080, phase I	Deliver large DNA, intrinsic tumor cell death capabilities, synergism with cargo	Transient gene expression, elicit an immune response, and tumor targeting capabilities are limited	
	Ad-p53	NCT00004041, phase I			
	AdV-tk	NCT00589875, phase IIa			
	AdV-tk	NCT00751270, phase I			
Herpes simplex virus	HSV1716 - replication restricted oncolytic herpes simplex virus with antitumor effects in multiple cell lines		Demonstrated safety in the clinic	Limited distribution within tumor	
	C134 - genetically engineered herpes simplex virus				
					G207 - neuroattenuated, replication-competent, recombinant herpes simplex virus-1	
	rQNestin34.5v.2 - an oncolytic viral vector made from the herpes simplex virus type 1				

Table 6 summarizes the advantages and limitations of the nonviral vectors used for gene therapy.

**Table 6:** Advantages and limitations of the nonviral vectors used for gene therapy

Vector	Clinical trial number and phase	Advantages	Disadvantages
Gold nanoparticles - NU-0129	NCT03020017, early phase I	Multimodal use for tumor imaging and therapy, ability to functionalize for targeting	Nonbiodegradable, trafficking the tumor tissue can be inefficient
Dendrimer and dendrigraft		Self-assemble with nucleic acids	Increased cytotoxicity for cationic dendrimers
		Ability to functionalize for targeting	Limited release of therapeutics
		Non-immunogenic	
Polymeric micelles		Self-assemble with nucleic acids	Increased cytotoxicity
		Ability to functionalize for targeting	Low loading efficiency
Poly (β-amino ester)		Biodegradable	Limited control over the release of therapeutics
		Lower cytotoxicity than other cationic polymers	
		High transfection efficiency	

Table 7 summarizes the advantages and limitations of tumor-tropic cell carriers expressing therapeutic gene(s) in the tumor site.

**Table 7:** Advantages and limitations of tumor-tropic cell carriers expressing therapeutic gene(s) in the tumor site BBB: blood-brain barrier

Vector	Advantages	Disadvantages	
Neural stem cells	Multiple administration routes are possible	Genetic material can be toxic to stem cells	
Mesenchymal stem cells	Traffic efficiently to the brain	Can be rejected by the immune system if not autologous	
	Can carry therapeutics, including viruses, across the BBB	Possibility of tumor formation	
Intelligent carriers			
			pH-sensitive drug release	Temporal release of therapeutics prevents toxicity to surrounding tissues	Research in its infancy	
			pH-sensitive liposomal carriers	Extensive modification possible	The efficiency of intelligent release in vivo is still uncertain	
Stimuli-responsive particles	Can carry therapeutics across the BBB		

Oncolytic Virotherapy (OV)

**Figure 3:**
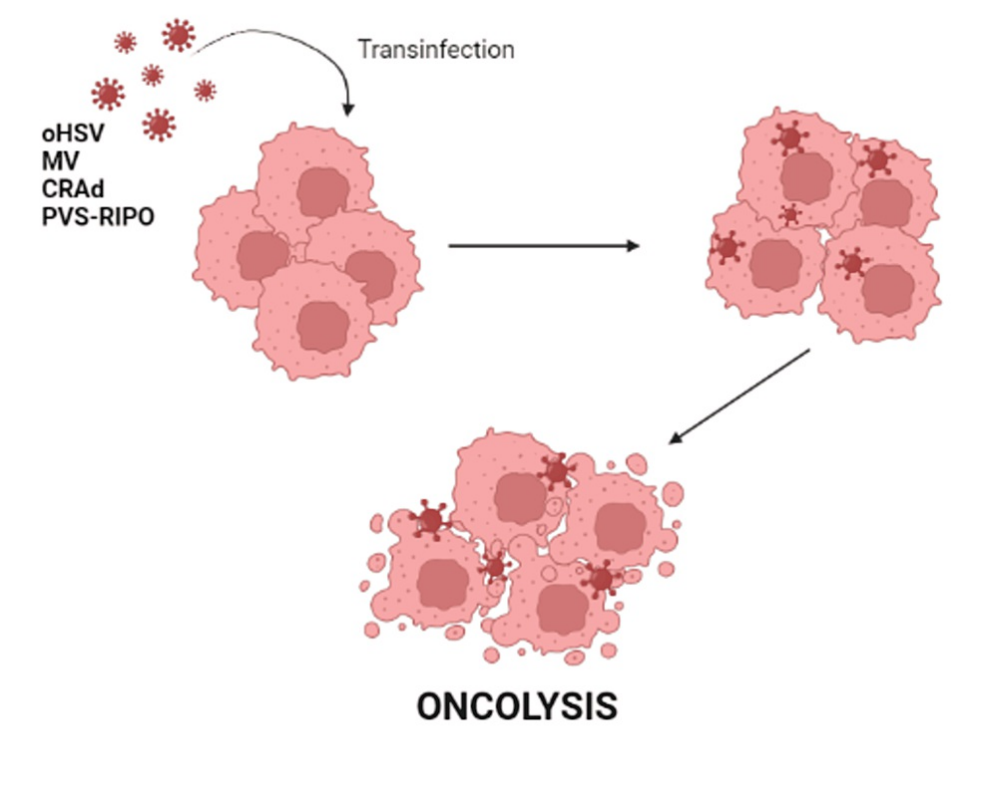
Mechanism of action of oncolytic viruses CRAd: conditionally replicating adenovirus; PVS-RIPO: recombinant nonpathogenic poliorhinovirus; MV: measles virus; oHSV: oncolytic herpes simplex virus This figure was originally created by the author.

OVs are intended to particularly infect cancer cells, self-replicate, induce oncolysis, and amplify therapeutic genes at tumor sites [27]. The advantages of OV include its high transduction efficiency and intra-tumoral viral spread, the capability of producing high viral titers, accessibility to genetic engineering, and adding additional therapeutic transgenes. Its limitations include host immune rejection/suppression of the virus, safety risks surrounding replication-competent viruses, and requirement of local administration during surgery [30]. Figure 3 explains the mechanism of action of oncolytic virotherapy.

Oncolytic herpes simplex virus (oHSV) are double-stranded deoxyribonucleic acid (DNA) viruses, a human pathogen with neurotropic properties. The challenge in designing oHSVs is to provide tumor selectivity while maintaining an acceptable safety profile [27]. Early clinical trial results showed that numerous oHSV vectors had high safety profiles with no signs of encephalitis but poor therapeutic effectiveness [31].

Conditionally replicating adenovirus (CRAd) are nonenveloped DNA viruses capable of infecting both the dividing and nondividing cells. An important advantage of CRAd viruses is that they are naturally non-neurotropic and have an enhanced safety profile over the oHSV vector. ONYX-015 and Ad5-Delta24 bare the most widely studied CRAd [14]. ONYX-015 contains a deletion in the viral protein early region 1B-55K (E1B-55K), which normally binds to and inactivates the host cell p53 protein. Therefore, it is assumed that cells with functional p53 cannot support viral replication in the absence of this protein, whereas tumor cells with a nonfunctional support viral replication.

Oncolytic measles, reovirus vectors, and recombinant nonpathogenic polio rhinovirus (PVS-RIPO) are reoviruses that only replicate in glioma cells because platelet-derived growth factor receptor (PDGFR) or EGFR stimulation of the KRAS pathway suppresses ribonucleic acid (RNA)-activated protein kinase activation. Clinical trial demonstrates that they are safe and well-tolerated with no evidence of clinical encephalitis. Measles virus (MV) exhibits the mutated hemagglutinin envelope glycoprotein H, which targets the cluster of differentiation 46 (CD46) on glioma cells. The circulating carcinogenic embryonic antigen (CEA) was modified into MV, which can be used to measure virus replication and oncolytic function [27]. PVS-tumor RIPO’s cell tropism is determined by the poliovirus receptor CD155, which is expressed on high-grade glioma cells. The clinical trials’ findings revealed satisfactory antitumor effectiveness but a low safety profile. Table 8 summarizes clinical trials and results on oncolytic virotherapy.

**Table 8:** Clinical trials and results on oncolytic virotherapy HSV: herpes simplex virus; PVSRIPO: recombinant nonpathogenic poliorhinovirus; HGG: high-grade glioma; G207: neuroattenuated, replication-competent, recombinant herpes simplex virus-1; HSV1716: replication restricted oncolytic herpes simplex virus with antitumor effects in multiple cell lines; DNX-2401 (tasadenoturev): a tumor-selective, replication-competent oncolytic adenovirus

ClinicalTrials.gov identifier	Title	Result	Phase, status, and number of patients enrolled	Diseases	
NCT00028158	Safety and Effectiveness Study of G207, a Tumor-Killing Virus, in Patients with Recurrent Brain Cancer	There are few side effects and a synergistic effect with concurrent radiotherapy, but efficacy remains limited	I/II, completed, 65	Glioma, astrocytoma, glioblastoma	
NCT00157703	G207 Followed by Radiation Therapy in Malignant Glioma	There are few side effects and a synergistic effect with concurrent radiotherapy, but efficacy remains limited	I, completed, 9	Malignant glioma	
NCT02031965	Oncolytic HSV-1716 in Treating Younger Patients With Refractory or Recurrent High-Grade Glioma That Can Be Removed by Surgery	Good tolerance, the major weakness lies in the deletion of γ34.5, which reduces viral activity and efficacy	I, terminated, 1	Brain and central nervous system tumors	
					NCT02197169	DNX-2401 With Interferon Gamma (IFN-γ) for Recurrent Glioblastoma or Gliosarcoma Brain Tumors	No significant difference in survival was reported between the two groups	I, completed, 37	Glioblastoma, gliosarcoma	
NCT00390299	Viral Therapy in Treating Patients with Recurrent Glioblastoma Multiforme	No severe side effects were reported	I, completed, 23	Anaplastic astrocytoma, anaplastic oligodendroglioma, mixed glioma, recurrent glioblastoma	
NCT02062827	Genetically Engineered HSV-1 Phase 1 Study for the Treatment of Recurrent Malignant Glioma	Showing relevant oncolytic activity against HGGs	I, recruiting, 36	Recurrent glioblastoma multiforme, progressive glioblastoma multiforme, anaplastic astrocytoma or gliosarcoma	
NCT03911388	HSV G207 in Children with Recurrent or Refractory Cerebellar Brain Tumors	There are few side effects and a synergistic effect with concurrent radiotherapy, but efficacy remains limited	I, recruiting, 15	Brain and central nervous system tumors	
NCT00805376	DNX-2401 (Formerly Known as Delta-24-RGD-4C) for Recurrent Malignant Gliomas	Median overall survival (OS) was 9.5 months and 13 months for group 1 and 2, respectively	I, completed, 37	Brain cancer, central nervous system diseases	
NCT02986178	PVSRIPO in Recurrent Malignant Glioma	Sufficient anticancer efficacy, but a low safety profile	II, active, not recruiting, 122	Malignant glioma	
					NCT03973879	Combination of PVSRIPO and Atezolizumab for Adults with Recurrent Malignant Glioma	Sufficient anticancer efficacy, but a low safety profile	I/II, withdrawn	Malignant glioma	
NCT03043391	Phase 1b Study PVSRIPO for Recurrent Malignant Glioma in Children	Sufficient anticancer efficacy, but a low safety profile	I, recruiting, 12	Brain and central nervous system tumors						
NCT02457845	HSV G207 Alone or With a Single Radiation Dose in Children With Progressive or Recurrent Supratentorial Brain Tumors	There are few side effects and a synergistic effect with concurrent radiotherapy, but efficacy remains limited	I, active, not recruiting, 12	Brain and central nervous system tumors, head and neck cancer, oropharyngeal cancer	

Suicide Gene Therapies 

**Figure 4:**
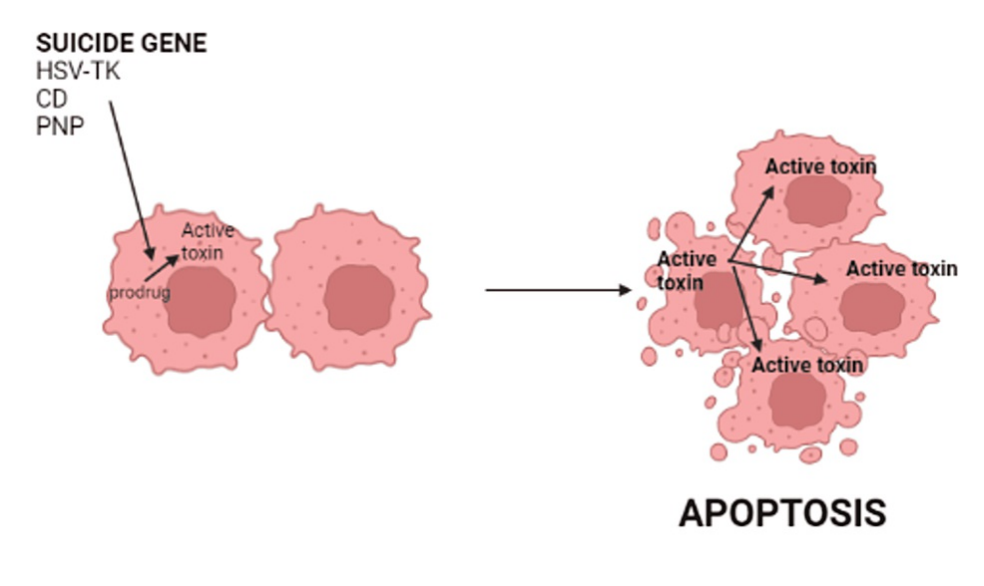
Mechanism of action of suicide gene therapy CD: cytosine deaminase; PNP: *E. coli*-derived purine nucleoside phosphorylase; HSV-tk: herpes simplex virus thymidine kinase This figure was originally created by the author.

The suicide gene technique is based on virally delivering “suicide genes” to target cells, which produce enzymes that convert prodrugs to active compounds. The inert prodrug is given systematically and then activated by suicide enzymes at the tumor site, resulting in tumor cell apoptosis [27]. Its advantages include achieving a “bystander effect,” requiring short-term gene expression, selective tumor cell targeting, and enhancing sensitivity to conventional therapy. It is restricted by the limited spatial distribution of gene transfer vectors, poor gene transfer efficiency into tumor cells in vivo, inability to target dispersed tumor cells, and restricted intra-tumoral distribution. Figure 4 explains the mechanism of action of suicide gene therapy.

Herpes simplex virus thymidine kinase (HSV-tk) enzyme catalyzes ganciclovir/valacyclovir monophosphorylation, which occurs after the triphosphorylation and activation of intracellular kinases. The active medication inhibits DNA synthesis and tumor lysis by blocking the S phase and arresting the cell circle. Cytosine deaminase (CD) catalyzes the activation of the prodrug 5-fluorocytosine (5-FC). A replication-competent retrovirus called Toca 511 loads the CD and transinfects tumor cells. It stimulates the expression of CD, which activates the 5-FU, which blocks DNA synthesis irreversibly and causes cell death. *Escherichia coli*-derived purine nucleoside phosphorylase (PNP) transforms adenosine ribonucleosides, such as fludarabine, into the active adenine molecule, 2-fluoroadenine, which disrupts RNA replication and the cell cycle. Antibiotic therapy, which suppresses intestinal flora, may over-activate the PNP gene therapy, resulting in increased prodrug conversion [27]. Table 9 summarizes clinical trials and results on suicide gene therapies.

**Table 9:** Clinical trials and results on suicide gene therapies HSV-tk: herpes simplex virus thymidine kinase; SBRT: stereotactic body radiation therapy; CMV: cytomegalovirus; GBM: glioblastoma multiforme; GCV: ganciclovir; XRT:- radiotherapy; Adv-tk: adenovirus-mediated herpes simplex virus thymidine kinase; 5-FC: 5-fluorocytosine; TOCA511: retroviral replicating vector that selectively infects cancer cells and delivers cytosine deaminase

ClinicalTrials.gov identifier	Title	Results	Phase, status, number of patients enrolled	Diseases
NCT03596086	HSV-tk + Valacyclovir + SBRT + Chemotherapy for Recurrent GBM	Results demonstrated the safety of this strategy with promising antitumoral efficacy	I/II, recruiting, 62	Glioblastoma multiforme, astrocytoma grade III
NCT00589875	Phase 2a Study of AdV-tk with Standard Radiation Therapy for Malignant Glioma (BrTK02)	Results demonstrated the efficiency in the use of adenovirus as the carrier	II, completed, 52	Malignant glioma, glioblastoma multiforme, anaplastic astrocytoma
NCT03603405	HSV-tk and XRT and Chemotherapy for Newly Diagnosed GBM	Results demonstrated the safety of this strategy with promising antitumoral efficacy	I/II, recruiting, 62	Glioblastoma, anaplastic astrocytoma, neoplasm metastasis
NCT01470794	Study of a Retroviral Replicating Vector Combined with a Prodrug to Treat Patients Undergoing Surgery for a Recurrent Malignant Brain Tumor	Results showed a good safety profile and a median overall survival of 12-14 months	I, completed, 58	Glioblastoma multiforme, anaplastic astrocytoma, anaplastic oligodendroglioma, anaplastic oligoastrocytoma
NCT00390299	Viral Therapy in Treating Patients with Recurrent Glioblastoma Multiforme	No severe side effects were reported	I, completed, 23	Anaplastic astrocytoma, anaplastic oligodendroglioma, mixed glioma, recurrent glioblastoma
NCT02414165	The Toca 5 Trial: Toca 511 & Toca FC Versus Standard of Care in Patients with Recurrent High-Grade Glioma	Therapeutic failure of Toca 511/5-FC compared to the standard of care	II/III, terminated, 403	Glioblastoma multiforme, anaplastic astrocytoma

Tumor Suppressor Gene Therapies 

**Figure 5:**
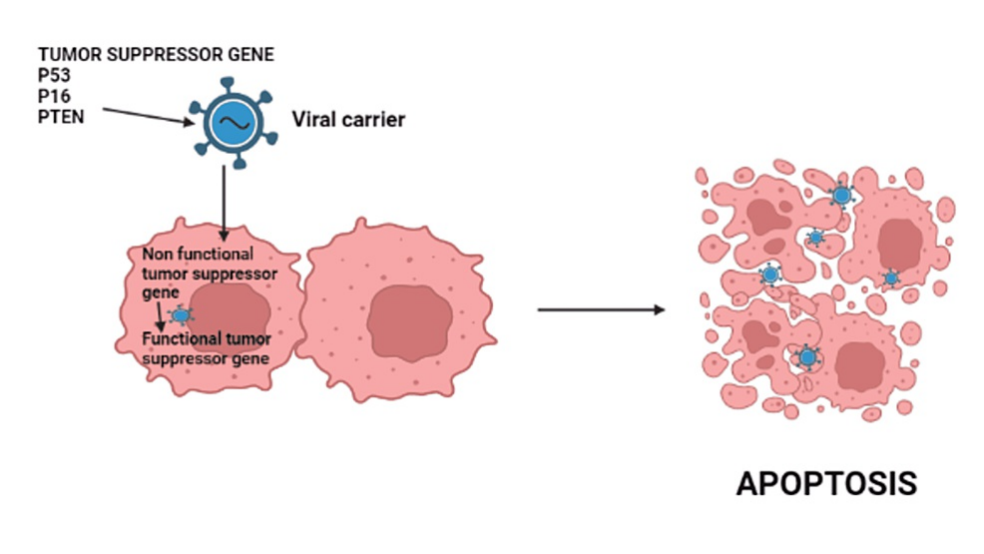
Mechanism of action of tumor suppressor gene therapy PTEN: phosphatase and tensin homologs This figure was originally created by the author.

High-grade gliomas frequently have deletions and mutations in tumor suppressor genes such as p53, p16, and phosphatase and tensin homologs (PTEN) [2]. Tumor suppressor gene techniques aim to restore normal function by transferring antitumoral functional genes to glioma cells. The advantages are safety in clinical trials, the potential to induce senescence within tumors, and the potential to sensitize tumor cells to other therapies. The limitations are as follows: multiple redundant pathways in tumors hinder efficacy, poor in vivo gene transfer, and limited distribution of therapy. Figure 5 explains the mechanism of action of tumor suppressor gene therapy.

P53 is involved in the inhibition of angiogenesis and DNA repair pathways. E1 gene is replaced by wild-type p53 in adenovirus and transmitted via a cytomegalovirus promoter (Ad5CMV-p53), which is the most widely used method. The E1 deletion prevents the virus from starting the infectious phase, while the cytomegalovirus promoter boosts the production of the p53 gene [27].

P16 prevents uncontrolled replication and oncogenesis by arresting the cell cycle during the G1-S transition [32]. Restoration of p16 function through an adenoviral vector has been found to decrease glioma growth and locoregional dissemination while also inhibiting matrix metalloprotease activity in the glioma microenvironment [33]. The adenovirus-mediated p16 gene was used to drive p16-null human glioma cell lines to enter phase G1 of the cell cycle. In HGG cells, data revealed that p16 expression is linked to tumor radiosensitivity through mechanisms of aberrant nucleation [34]. It is worth noting that the efficiency of the p16 gene approach is contingent on maintaining retinoblastoma protein (pRB) activity [35].

The PTEN gene has been shown to suppress glioma proliferation and induce oncolysis when delivered through an adenoviral vector [27]. Adenoviral vector transfer of the PTEN gene into glioma cells improved tumor sensitivity to temozolomide and radiation [36]. Table 10 summarizes clinical trials and results on tumor suppressor gene therapies.

**Table 10:** Clinical trials and results on tumor suppressor gene therapies PFS: progression-free survival; OS: overall survival; CNS: central nervous system

ClinicalTrials.gov identifier	Title	Results	Phase, status	Diseases
NCT00004041	Gene Therapy in Treating Patients With Recurrent Malignant Gliomas	Progression-free survival (PFS) of 13 weeks and OS of 44 weeks	Phase I, completed	Brain and CNS tumors
NCT00004080	Gene Therapy in Treating Patients With Recurrent or Progressive Brain Tumors	PFS of 13 weeks and OS of 44 weeks	Phase I, completed	Brain and CNS tumors

Immunomodulatory Gene Therapies 

**Figure 6:**
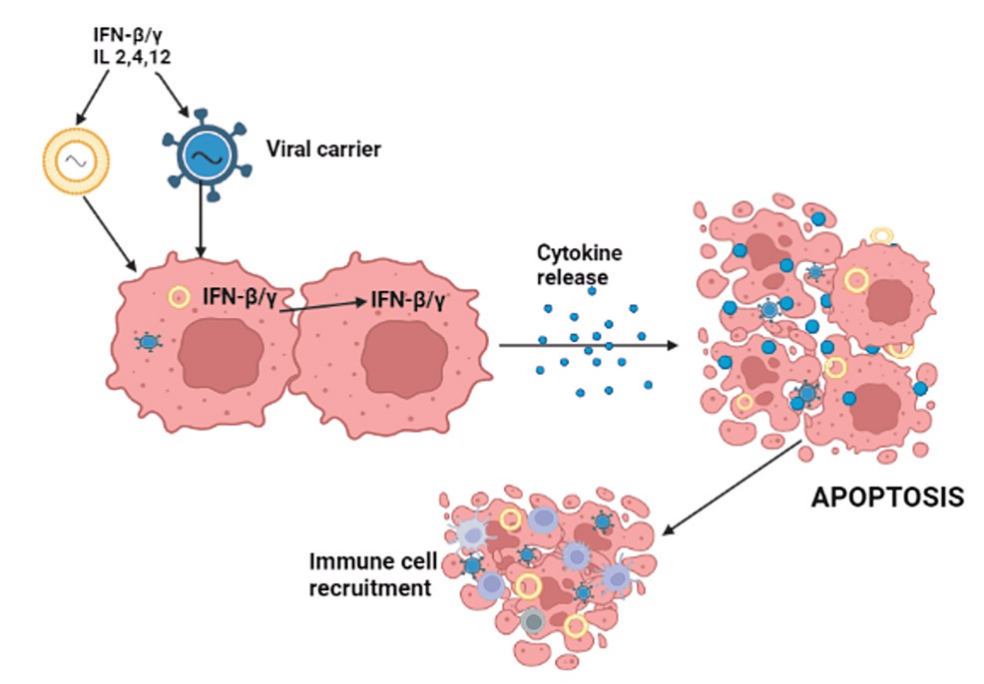
Mechanism of action of immunomodulatory gene therapy IFN-β/γ: interferon beta/gamma; IL: interleukin This figure was originally created by the author.

The objective of anti-glioma immunomodulatory gene therapy is to induce or augment the T-cell-mediated immune response against tumors using the delivery of genes for immunostimulatory cytokines and interferon beta/gamma (IFN-β/γ) [27]. Its advantages include the following: this therapy can achieve passive or active tumor immunity, it has the possibility to eliminate tumor cells that remain post-surgery, and it regulates the tumor microenvironment. This therapy is limited by tumor-induced immunosuppression, lack of antigen-presenting dendritic cells within the brain, and overcoming the presence of immune-suppressive regulatory T-cells and cytokines. Figure 6 explains the mechanism of action of immunomodulatory gene therapy.

The stimulation of natural killer cells and macrophages demonstrated potential antitumoral action [37]. INF-β was also transferred using nanoparticles and liposomes. Clinical trial shows a reduction in volumetric glioma and mild toxicity [38]. Histological findings reported an elevated level of immune activation [39]. IFN-β inhibits cancer cell proliferation and interactions with the extracellular matrix [40].

Interleukin-12 (IL-12) is one of the most important immunostimulant cytokines for strengthening the immune system and attracting cytotoxic cells in the tumor microenvironment. Nonreplicating adenoviruses and HSV were used in an earlier phase of research to deliver IL-12 to malignant glioma cells. Preclinical research revealed tumor cell death, active microglia cell infiltration, a favorable safety profile, and a significant local immune response [27].

Several clinical trials have shown that chemotherapy has a synergistic impact when combined with immunotherapy, challenging the conventional dogma that chemotherapy-induced immunosuppression prevents the formation of antitumor immune responses. In a limited phase I clinical trial, three pediatric patients with recurring brain tumors were given a combination of high-dose chemotherapy and adoptive immunotherapy [41]. Accumulating preclinical and clinical evidence suggests that combining tumor cell killing techniques with immunotherapy results in synergism between the two therapies, resulting in improved efficacy and lower toxicity. This collection of evidence refutes the conventional notion that tumor cell killing tactics hinder the immune system’s ability to recognize and eradicate a brain tumor, and it supports the use of combined cytotoxic-immunotherapeutic strategies in the treatment of glioblastoma multiforme patients [42]. Table 11 summarizes clinical trials and results on immunomodulatory gene therapy.

**Table 11:** Clinical trials and results on immunomodulatory gene therapy Ad-RTS-hIL-12: inducible adenoviral vector engineered to express IL-12; DIPG: diffuse intrinsic pontine glioma; IFN: interferon; NK: natural killer; T-cells: T lymphocytes

ClinicalTrials.gov identifier	Title	Results	Phase, status, number of patients enrolled	Diseases
NCT00031083	Dose Escalation Study to Determine the Safety of IFN-Beta Gene Transfer in the Treatment of Grade III & Grade IV Gliomas	The findings supported the activation of the immune cascade and the recruitment of T and NK cells in the tumor microenvironment	I, completed, 12	Glioblastoma multiforme, anaplastic astrocytoma, oligoastrocytoma, mixed gliosarcoma
NCT02026271	A Study of Ad-RTS-hIL-12 With Veledimex in Subjects with Glioblastoma or Malignant Glioma	The study discovered a significant increase in antitumor infiltrating lymphocytes	I, active, 48	Glioblastoma multiforme, anaplastic oligoastrocytoma, pediatric brain tumor
NCT03330197	A Study of Ad-RTS-hIL-12 + Veledimex in Pediatric Subjects with Brain Tumors Including DIPG	The study discovered a significant increase in antitumor infiltrating lymphocytes	I/II, recruiting, 45	Diffuse intrinsic pontine glioma

Gene Target Therapies 

**Figure 7:**
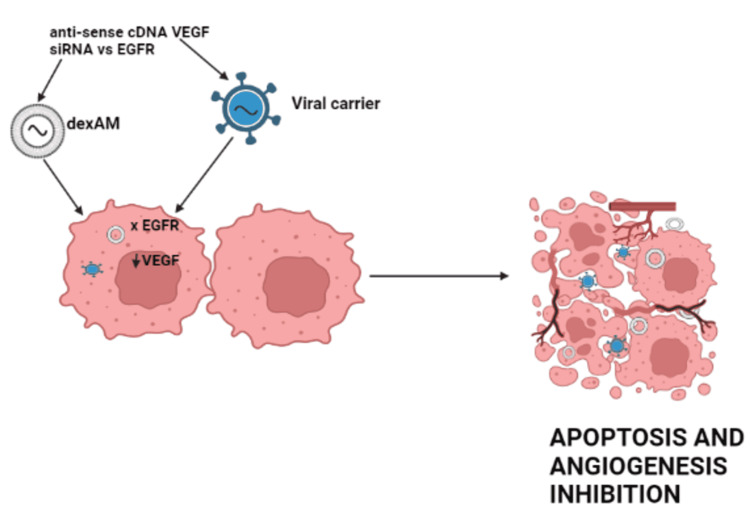
Mechanism of action of target gene DexAM: cyclodextrin-modified dendritic polyamine complexes; EGFR: epidermal growth factor receptor; VEGF: vascular endothelial growth factor; cDNA: complementary deoxyribonucleic acid; siRNA: small interfering ribonucleic acid This figure was originally created by the author.

Gene target medicines directly bind specific tumor antigens to block oncogenic pathways irreversibly. Figure 7 explains the target gene mechanism of action.

The epidermal growth factor receptor (EGFRvIII) variation, which is prevalent in 30% of high-grade gliomas, is involved in oncogenesis and tumor development processes. Antisense or short interfering RNA (siRNA) directed exclusively targeting the thymidine kinase domain of glioma EGFRvIII was delivered by viral vectors and nanoparticles [43]. The delivery of EGFRvIII siRNA using cyclodextrin-modified dendritic polyamine complexes (DexAMs) exhibited promising effects in malignant glioma cells, even when combined with erlotinib [44].

Direct intra-tumoral inoculation of polyethylenimine (PEI)/VEGF siRNA had a substantial antiangiogenic impact on xenografts [44]. In the Matrigel plug experiment, Ad-DeltaB7-shVEGF, an adenovirus construct, was developed, expressing a short hairpin RNA against VEGF; it showed excellent antiangiogenic action and better bioavailability than replication-incompetent adenoviruses [45]. In a human xenografted glioma model, Ad-DeltaB7-KOX, an oncolytic adenovirus, showed strong anticancer efficacy [46]. Another study looked at HGGs infected with adenovirus expressing vascular endothelial growth factor receptor (VEGFR) and the oncolytic virus dl922/947. This combination therapy was more successful than monotherapy [27].

## Conclusions

Despite recent advances in surgery, radiotherapy, and chemotherapy, brain tumor treatment regimens have only a limited impact on long-term disease control. Requirement for the development of novel treatments such as gene therapy arose over the past decades. We still have a long way to go before we can honestly say that gene therapy for pediatric cancer has had a significant impact on these diseases, but gene therapy can improve standard treatment efficacy when used as adjuvant therapy. It can be used to overcome barriers such as chemotherapy resistance by downregulating resistance genes or using approaches such as suicide gene therapy. Gene therapy is a better option in this age of precision medicine, and although the phase three clinical study lacks gene therapy advancements, it can make a drastic improvement in brain tumor treatment. It is associated with mild toxicity compared with other available treatment options, and given the overall poor prognosis in pediatric brain tumors, further studies are warranted.
